# Supplementary material for: k-mer manifold approximation and projection for visualizing DNA sequences
Source: Genome Res. 2025 May;35(5):1234–46. doi: 10.1101/gr.279458.124 (PMC12047656; doi:10.1101/gr.279458.124)
Supplement: Supplement 8 [file Supplemental_Data_1.zip › Supplemental_Data_1/HLF_TAGCCT20NCG_AD_4/HLF_TAGCCT20NCG_AD_4_tSNE.pdf]

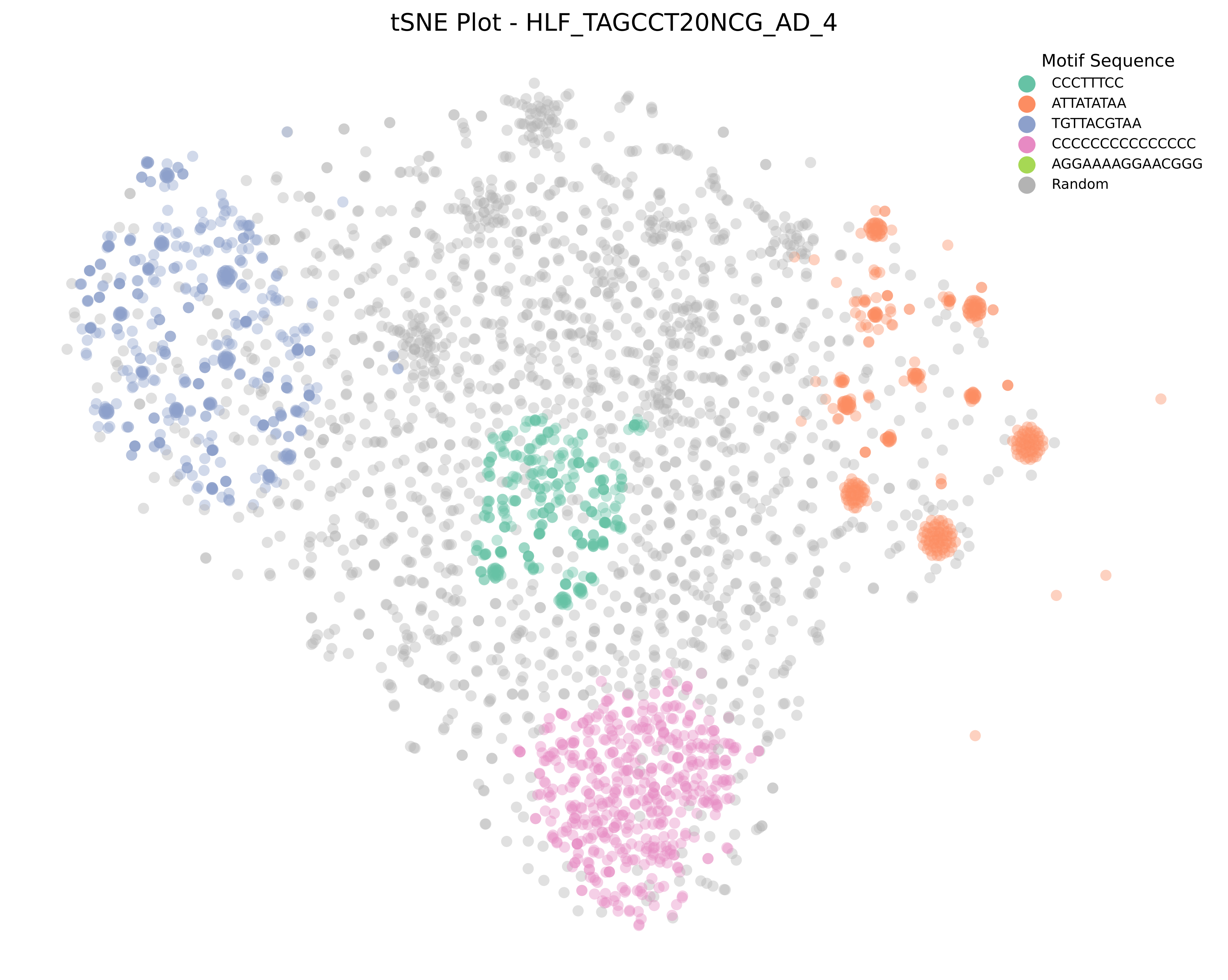
tSNE Plot - HLF_TAGCCT20NCG_AD_4
Motif Sequence
CCCTTTCC
ATTATATAA
TGTTACGTAA
CCCCCCCCCCCCCCC
AGGAAAAGGAACGGG
Random
